# Are Inflamed Periodontal Tissues Endogenous Source of Advanced Glycation End-Products (AGEs) in Individuals with and without Diabetes Mellitus? A Systematic Review

**DOI:** 10.3390/biom12050642

**Published:** 2022-04-27

**Authors:** Aditi Chopra, Thilini N. Jayasinghe, Joerg Eberhard

**Affiliations:** 1 Department of Periodontology, Manipal College of Dental Sciences, Manipal Academy of Higher Education, Manipal 576104, India; 2 The Charles Perkins Centre, The University of Sydney, Sydney, NSW 2006, Australia; thilini.jayasinghe@sydney.edu.au (T.N.J.); joerg.eberhard@sydney.edu.au (J.E.); 3 School of Dentistry, Faculty of Medicine and Health, The University of Sydney, Sydney, NSW 2006, Australia

**Keywords:** periodontitis, diabetes mellitus, inflammation, hyperglycemia, oral health, oxidative stress, advanced glycation end-products, biomolecules

## Abstract

Advanced glycation end-products (AGEs) are heterogeneous compounds formed when excess sugars condense with the amino groups of nucleic acids and proteins. Increased AGEs are associated with insulin resistance and poor glycemic control. Recently, inflamed periodontal tissues and certain oral bacteria were observed to increase the local and systemic AGE levels in both normoglycemic and hyperglycemic individuals. Although hyperglycemia induced AGE and its effect on the periodontal tissues is known, periodontitis as an endogenous source of AGE formation is not well explored. Hence, this systematic review is aimed to explore, for the first time, whether inflamed periodontal tissues and periodontal pathogens have the capacity to modulate AGE levels in individuals with or without T2DM and how this affects the glycemic load. Six electronic databases were searched using the following keywords: (Periodontitis OR Periodontal disease OR Periodontal Inflammation) AND (Diabetes mellitus OR Hyperglycemia OR Insulin resistance) AND Advanced glycation end products. The results yielded 1140 articles, of which 13 articles were included for the review. The results showed that the mean AGE levels in gingival crevicular fluid was higher in individuals with diabetes mellitus and periodontitis (521.9 pg/mL) compared to healthy individuals with periodontitis (234.84 pg/mL). The serum AGE levels in normoglycemic subjects having periodontitis was higher compared to those without periodontitis (15.91 ng/mL vs. 6.60 ng/mL). *Tannerella* *forsythia*, a common gram-negative anaerobe periodontal pathogen in the oral biofilm, was observed to produce methylglyoxal (precursor of AGE) in the gingival tissues. Increased AGE deposition and activate of AGE receptors was noted in the presence of periodontitis in both normoglycemic and hyperglycemic individuals. Hence, it can be concluded that periodontitis can modulate the local and systemic levels of AGE levels even in absence of hyperglycemia. This explains the bidirectional relationship between periodontitis and development of prediabetes, incident diabetes, poor glycemic control, and insulin resistance.

## 1. Introduction

Periodontitis is a chronic inflammatory disease of the soft tissue surrounding the teeth, affecting nearly 20–50% of adults globally [1,2,3,4]. The main etiology of periodontitis is the interaction of the host with the microorganisms in the oral biofilm. *Porphyromonas gingivalis, Streptococcus sanguis*, *Actinomyces viscosus*, *Tannerella forsythia*, *Fusobacterium nucleatum*, and *Treponema denticola* are some of the common microorganisms linked with the onset and progression of periodontal disease [5,6]. The interaction between these microorganisms and the host activates a series of immune and inflammatory responses that cause a massive release of various chemical mediators of inflammation, immune cells, proteolytic enzymes, microbial products, and free radicals or reactive oxygen species (ROS) in the gingival and periodontal tissues [7,8,9,10]. The influx of these inflammatory mediators and immune cells mark the onset of periodontal disease, also referred to as gingivitis [8]. Gingival bleeding, redness of the gingiva, gingival enlargement, and an increase in the gingival crevicular fluid (GCF) flow are common clinical signs of gingivitis. If untreated, gingivitis progresses to periodontitis, which clinically manifests as pocket formation, loss of connective tissue attachment, tooth mobility, gingival recession, alveolar bone loss, and subsequently loss of teeth [4,8]. Thus, it can conclude that the rate of the progression and severity of periodontal inflammation are related to the host’s inflammatory response, oxidative stress, and amount of microbial load in the gingival and periodontal tissues. However, various environmental and systemic factors—such as smoking [11,12,13], type 2 diabetes mellitus (T2DM) [14,15,16,17], immunocompromised disease conditions (e.g., HIV) [18,19], nutritional deficiency [20,21,22], medications (e.g., drug-induced gingival overgrowth) [23,24], poor oral hygiene, and genetic factors [25]—influence the progression of gingival and periodontal inflammation.

Periodontitis induced inflammatory response and oxidative stress can increase the oxidative stress and the inflammatory burden in the systemic circulation affecting distant organ systems [26]. Periodontitis has been linked with many systemic diseases, such as cancer, cardiovascular diseases, respiratory tract infections, chronic kidney diseases, adverse pregnancy outcomes, neurodegenerative disease, T2DM, prediabetes, gestational diabetes, and incident diabetes [27,28,29,30,31,32]. The link between periodontitis and systemic disease is related to the ability of periodontal inflammatory mediators to enter the systemic circulation and activation of acute-phase response with the release of various proteins such as C-reactive proteins (CRPs), cytokines, and ROS [26]. These interactions of these mediators with cells and receptors in body increase the systemic oxidative stress, induce dysfunction and apoptosis of cells and tissues, cause atherosclerotic changes in the vasculature, and alter the body’s metabolism [9,28].

**Figure 1:**
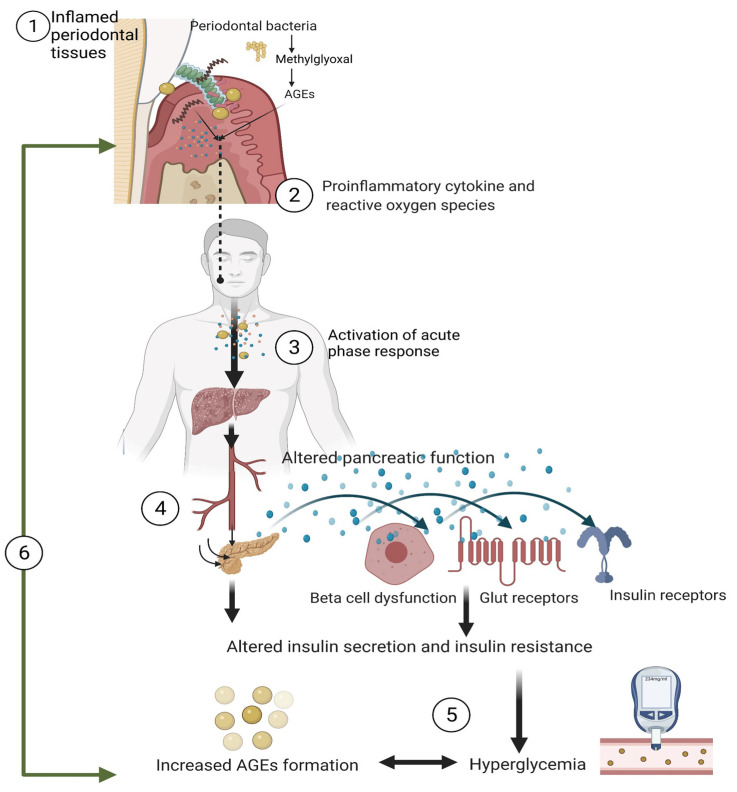
Schematic representation explaining how periodontitis can increase AGE levels.

Periodontitis-induced oxidative stress is known to play an important role in ‘hyperglycemia-induced tissue injury’ and in early events related to the onset of T2DM and its complications [9,33]. Periodontitis is even confirmed to have a bidirectional relationship with diabetes mellitus (DM) [34]. Studies have confirmed that individuals with diabetes have a 2.8- to 3.4-times higher risk of having periodontitis compared to healthy subjects [35,36]. Individuals with severe periodontitis have a six-fold increase in the risk of worsening glycemic control over time than those without periodontitis [37]. Additionally, the presence of severe periodontitis is associated with poor diabetic management and vice-versa [14,38,39]. Studies have shown that patients with periodontitis have higher circulating white blood cells, acute-phase proteins (CRPs), tumor necrosis factor (TNF), interleukins (IL), and ROS [40]. TNF, interleukin 1 (IL1), IL6, IL17, prostaglandin E2, and CRPs have been identified as risk factors for βeta cell dysfunction, insulin resistance, impaired glucose uptake, increased HbA1c levels, and worsening glycemic control in both normoglycemic and hyperglycemic individuals (Figure 1) [9,40,41,42,43,44,45]. A study by Auito et al. (2018) also showed that intensive periodontal treatment can improve the metabolic control in patients with T2DM along with the reductions in HbA1C and fasting plasma glucose [40]. Periodontal therapy can improve the vascular and kidney function, reduced systemic inflammation, and improve the quality of life. This evidence confirms a causal relation between periodontitis and diabetes control and the role of periodontitis as risk factor for diabetic complications [40].

(1) Periodontitis is caused by the interaction of the host with microorganisms in the oral biofilm. The host–microbial interaction causes a massive release of pro-inflammatory mediators (cytokines) and reactive oxygen species (ROS) in the gingival and periodontal tissues. Periodontal bacteria utilize the free glucose in the oral fluids and increase the production of the methylglyoxal synthase, an enzyme which catalyzes the formation of methylglyoxal, a precursor for advanced glycosylation end products (AGEs) and thereby increase the oxidative stress. (2) The locally produced pro-inflammatory chemical mediators, ROS, and microbial by-products along with AGEs enter the liver and activate the acute phase response that cause release numerous acute-phase proteins (such as C-reactive proteins). (3) The pro-inflammatory cytokines, acute phase proteins, and microbial products enter the pancreatic tissues and cause pancreatic dysfunction. (4) The cytokines—particularly interleukin 6, interleukin 12, and C reactive proteins—affect the insulin receptors, GLUT receptors, beta cells of the pancreas; reduce insulin secretion and increase insulin resistance which affect glucose uptake by cell and leads to hyperglycemia. (5) Reduced glucose uptake by the cell causes the excess glucose leading to glycation of host proteins and lipids, thereby forming more AGEs. (6) The increased AGEs levels interact with the insulin receptors, causing reduced insulin secretion and insulin resistance. The increase in the serum AGE levels via a vicious cycle increases oxidative stress, which, in turn, increases the severity of periodontal inflammation.

Apart from the inflammatory markers and oxidative stress, inflamed periodontal tissues have been shown to affect the glycemic load by modulating the AGE levels [46,47]. AGEs are heterogeneous compounds usually formed endogenously when excess sugars condense with proteins and nucleic acids to form an unstable aldimine intermediate or a Schiff base. The common AGEs produced in the systemic circulation are methylglyoxal, crossline, pentosidine, Nε-carboxyethyl-lysine (CEL), carboxymethyl-lysine (CEL), glyoxal-lysine-dimer (GOLD), methylglyoxal-lysine-dimer (MOLD), and pentosidine [48,49]. The increased deposition of AGEs is associated with various macrovascular and microvascular complications of DM and insulin resistance [42]. Although AGEs are formed endogenously in all individuals from the free glucose in the body (hyperglycemia) [33], recent studies have observed an increase in the AGE levels in saliva, GCF, serum, and gingival tissues in the presence of periodontitis even in normoglycemic individuals [50,51,52,53,54]. Periodontal pathogens have also been linked to the formation of methylglyoxal, an important AGE precursor [55]. These findings point to the role of periodontitis as an endogenous source of AGEs [56,57,58,59]. In spite of numerous epidemiological studies confirming the relationship between periodontitis and T2DM, no review has yet explored the role of periodontitis as an endogenous source of AGEs formation. Hence, this systematic review aims to explore, for the first time.whether inflamed periodontal tissues and periodontal pathogens have the capacity to modulate local and systemic AGE levels in individuals with or without T2DM. This evidence is crucial as it would establish a new pathogenic mechanism confirming the bi-directional relationship between periodontitis, diabetes, prediabetes, and insulin resistance.

## 2. Methods

### 2.1. Protocol and Registration

This systematic review was registered at PROSPERO: CRD42021229395 and was conducted according to “Preferred Reporting Items for Systematic Reviews and Meta-analysis (PRISMA) guidelines” [60].

### 2.2. Focus Question

To generate the evidence and answer the question of whether or not subjects with or without T2DM (Population) having periodontitis (Exposure) have higher AGE levels in GCF, saliva, serum, and gingival tissues (Outcome) as compared to subjects with or without DM and no periodontitis (Comparison). The review also aims to evaluate whether periodontal tissues or periodontal pathogens are a local source of AGE synthesis and understand how periodontitis can modulate the AGEs formation.

### 2.3. Search Strategy, Information Sources, and Keywords

MEDLINE (PubMed), Scopus, EBSCO (dentistry and open science access), Cochrane database, Web of Sciences, and ClinicalTrial.org. were searched on 2 September 2020, and updated on February 2021. The following keywords and MeSH terms (*) were utilized for data collection: [((“Periodontitis”[All Fields] OR “Periodontal disease” OR “Chronic Periodontitis” OR” Adult periodontitis” OR “Periodontal Inflammation”) AND (“diabetes”[All Fields] OR “diabetes mellitus” OR “Type 2 DM” OR “Type 2 diabetes mellitus” OR “non-insulin-dependent diabetes mellitus” OR “Hyperglycemia” OR “Insulin resistance”)) AND (“Advanced glycation end products” OR “Advanced glycosylation end products” OR “Millard Reaction” OR “carboxymethyl lysine” OR “Pentosidine”). The grey literature (Google Scholar) was also searched for relevant articles. *The Journal of Clinical Periodontology*, *Journal of Periodontology*, *Journal of Periodontal Research*, and *Journal of Dental Research* were searched manually to check for any recent articles (articles in press). Articles written in English in the last 20 years (2000 to 2021) were included. The search results were transferred into the Mendeley Reference Manager (version 1.19.4, from Elsevier, accessed from Manipal, India), and duplicates were removed. The details of the search strings on the individual database are provided in Table 1.

### 2.4. Inclusion and Exclusion Criteria

All observational studies (cross-sectional, case-control, cohort) investigating the relationship between periodontitis, T2DM, and AGEs were included. Studies were included if they provided either qualitative or quantitative data on the following outcomes: (1) periodontal condition as measured by periodontal probing depth (PD in mm), or clinical attachment level or loss (CAL in mm), or bleeding on probing (BOP), as a percentage of the number of teeth, or radiographic bone loss (% or unit measurement), or gingival index (GI), or plaque index (PI) and; (2) AGE levels or its receptors (RAGEs) or soluble forms of RAGE (sRAGE) in the GCF, saliva, serum, gingival or periodontal tissues. All preclinical studies, in-vitro studies, studies assessing the use of any intervention (such as use of any form of medication/non-surgical/surgical periodontal therapy) for the management of periodontitis and T2DM, case reports, case series, editorials, book reviews, literature reviews, and letters to the editor were excluded. Studies that did not report any data on AGEs or their receptors or did not report any diagnostic criteria for periodontitis or T2DM were excluded.

### 2.5. Study Screening and Data Extraction

**Figure 2:**
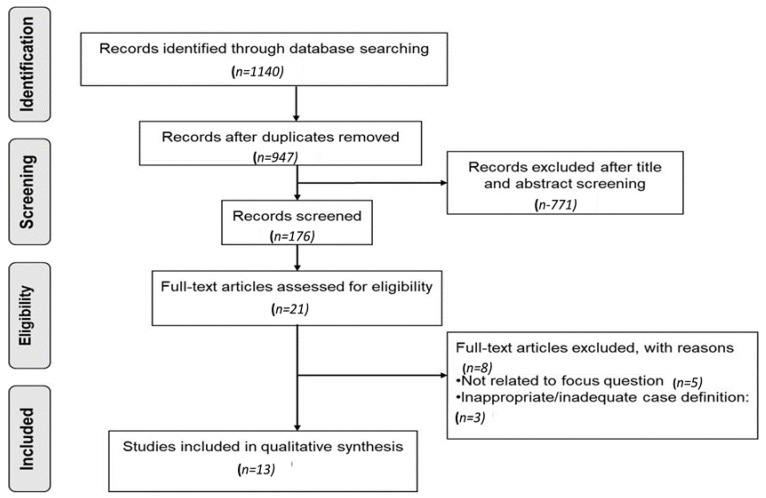
Prisma flow diagram.

The results from the data search were transferred into the Mendeley Reference (version 1.19.4, from Elsevier, accessed from Manipal, India), and the duplicates were removed. Two reviewers (A.C. and J.E) independently performed the data searches and the title and abstract screening based on the eligibility criteria. A.C and J.E made the final decision after mutually discussing any disagreements. The study selection process was reported by the Preferred Reporting Items for Systematic Reviews and Meta-Analyses (PRISMA) flow chart (Figure 2) [60]. Data extraction was performed on a pilot-tested spreadsheet by three authors independently. The following study characteristics were extracted:

1. Methods: trial design, duration of the study, country of the study, type of study design, and ethics committee approval (if mentioned).
2. Participants: number in each group, number analyzed, mean age, range, the gender of the participants; the severity of the condition, diagnostic criteria for diabetes and periodontitis (if mentioned).
3. Exposure: duration of periodontitis and diabetes (if mentioned).
4. The mean and standard deviation of the following outcomes:
  - Concentration of AGEs or receptors for AGE (RAGE) in any form in saliva/GCF/serum/blood/gingiva tissues.
  - Periodontal parameters: PD, CAL, BOP, GI, PI, the radiographic measure of bone loss (if present) as of all the outcomes.
  - Fasting, random, and postprandial blood glucose levels; glycated hemoglobin (whichever is applicable); body mass index (BMI); microbial profile; method of analysis of AGE.
5. Notes: Funding and potential conflicts of interest of authors in the study.

### 2.6. Risk of Bias (ROB) of Individual Studies

The quality assessment of full-text articles was conducted by two authors (A.C. and J.E.) by using the Newcastle–Ottawa Scale (NOS) [61]. All the studies were scored across three categories: <5 stars = high ROB; 6–7 stars = moderate ROB; and 8–9 stars = low ROB.

A meta-analysis was not performed because: (1) there was significant clinical heterogeneity among studies with regards to the diagnostic criteria for periodontitis and T2DM; (2) different methods used to analyze AGEs and their receptors (RAGEs); and (3) variation in the biologic samples used in the studies. The results are summarized as: (1) effect of periodontitis on the AGE levels and RAGE receptor activation in the gingiva tissues and oral fluids; and (2) effects of periodontitis on the serum AGEs and soluble forms of RAGE in the systemic circulation.

## 3. Results

### 3.1. Characteristics of the Included Studies

A total of 1140 articles were obtained from all databases (Figure 2 and Table 1). After removing the duplicates, 947 articles were retrieved and included for title and abstract screening. Upon title and abstract screening, 21 articles were included for full-text screening [52,53,54,55,56,57,59,62,63,64,65,66,67,68,69,70,71,72,73,74,75]. After the full-text screening, 8 articles were excluded [55,59,63,64,65,68,70,75], because they did not relate to our PICO/focus question and case definitions were inadequately reported (Supplementary Table S1). Therefore, a total of 13 studies were included for the review [52,53,54,56,57,58,66,67,69,71,72,73,74] (Table 2 and Table 3).

The quantitative or qualitative outcomes measured were: endogenously secreted AGE, soluble receptor for AGE (sRAGE) [52,67,74]; endogenous secretory RAGE (esRAGE) [52], Cleaved RAGE (cRAGE) [52]; and RAGE/AGE ratio. These outcomes were measured in saliva [54], GCF [71,72], serum [53,66,74], and gingival tissues [52,56,57,58,67,69,73]. AGE estimation was carried out quantitatively by ELISA [53,54,66,72,74] or qualitatively by immunohistochemistry [56,57,69,73]. The RAGE, cRAGE, and sRAGE were quantified by using RT-PCR, ELISA, immunohistochemistry, and spectrometry [52,56,57,67,69,71,73]. Two studies were rated as high risk of bias [69,73]; seven were rated with a moderate risk of bias [53,54,56,57,58,66,74] and four studies were rated as low risk of bias [52,67,71,72] (Table 3).The included studies were conducted in Germany [69], India [73,74], Iraq [53], Italy [58], Japan [66], Kingdom of Saudi Arabia [72], Taiwan [67], Turkey [54], and the USA [52,56,57,71] (Table 2). The sample size ranged from 7 to 230 participants [57,67]. All included studies used fasting blood glucose concentration and HbA1c levels to diagnose participants with T2DM. However, the threshold for the diagnosis of T2DM varied among studies (Table 2). The diagnostic criteria for the periodontal disease were PD, CAL, BOP, and radiographic bone loss (Table 2). Both males and females subjects were recruited, except for one clinical study on humans [53], where only male subjects were included.

### 3.2. Detection of AGEs and RAGE in Periodontal Tissues and Oral Fluids

Seven studies compared the AGEs in GCF, saliva, and gingival tissues in T2DM subjects with and without periodontitis compared to those without periodontitis [53,54,56,66,69,72,74]. The nature of AGEs was different as the severity of periodontal disease increased [66]. Methylglyoxal and Nε-carboxymethyl lysine were common in the early stages of periodontitis, while pentosidine, crossline, glyoxal-lysine dimer (GOLD), methylglyoxal-lysine dimer (MOLD), and imidazolines were more common in later stages of periodontitis [66]. The mean AGE levels in GCF were significantly higher in subjects with T2DM and periodontitis (521.9 pg/mL) compared to healthy individuals with periodontitis (234.84 pg/mL) or healthy individuals without periodontitis (87.2 pg/mL) with *p <* 0.01 [72]. The mean AGE level in saliva was also higher in subjects with T2DM and periodontitis (332  ±  350 ng/mL) compared to T2DM subjects without periodontitis (235  ±  360 ng/ml) with *p* < 0.001. Healthy subjects with periodontitis also showed more salivary AGE levels compared to healthy subjects without periodontitis (46.8  ±  52.1 ng/mL vs. 24.4  ±  38.5 ng/mL, respectively, *p*-value < 0.001) [54].

Immunohistochemistry analysis of gingival epithelium and connective tissues also revealed more AGE deposition and RAGE receptor activation in the presence of periodontitis [56,57,69,73]. A 1.3-fold increase in the percentage of AGE deposition was observed in the gingival connective tissues of subjects with periodontitis and T2DM (17%) compared to healthy controls with periodontitis (13%) [69]. The percentage of AGE-positive cells in the gingival epithelium was comparable in subjects with T2DM and periodontitis (75%) compared to healthy subjects with periodontitis (70%) and without periodontitis (62.5%) with *p* < 0.05 [57]. Moreover, AGE was noted around the gingival fibroblasts, and small blood vessels of the gingival connective tissue, spinous and basal layer of the inflamed gingival epithelium in subjects with periodontitis, and T2DM compared with healthy subjects without periodontitis [58]. AGE deposition was also increased in the periodontal ligament fibroblasts, inflammatory cell infiltrates, bone lining and perivascular cells, and macrophages in the presence of periodontitis [57,58,69,73]. *Tannerella forsythia*, a common periodontal pathogen, was reported to produce methylglyoxal, a precursor of AGEs in the gingival connective tissues. The methylglyoxal levels in GCF were found to be 94.2% higher in sites affected with periodontitis compared to healthy gingival tissues. The mean methylglyoxal levels in the GCF in subjects with periodontitis were 142.9 ± 235.7 pmol/site and 208. 7 ± 241.7 from periodontal pockets with less than 3 mm and those with more than 3 mm PD, respectively (*p* = 0.0023). The subjects without periodontitis and PD of less than 3 mm showed a mean methylglyoxal level of only 11.574.4 pmol/site [71].

The expression of AGE receptor (RAGE) was more around the inflammatory cells, endothelium, and epithelium in subjects with T2DM and periodontitis. The optical density (OD) for RAGE receptors was more in the gingival epithelium in subjects with T2DM and periodontitis (46.91 ± 5.57 nm) as compared to healthy patients with periodontitis (31.42 ± 7.42 nm) and healthy patients without periodontitis (21.54 ± 1.46 nm) (*p* < 0.001) [56]. However, when subjects with T2DM with either moderate or severe forms of periodontitis were compared, no statistically difference in the RAGE expression was noted either around the epithelial (*p* = 0.57) or inflammatory cells (*p* = 0.69). This indicated that the expression of RAGE in T2DM patients was not affected by the severity of periodontitis [56]. Similarly, the expression of mRNA for RAGE receptor was higher (50% more) in patients with T2DM and periodontitis compared to healthy patients without periodontitis (*p* < 0.05) [57]. Moreover, a statistically significant difference in the mRNA expression for RAGE was noted in subjects without T2DM as the severity of periodontitis increased (*p* < 0.001). This confirms that the expression of RAGE is affected by the severity of periodontitis, even in the absence of T2DM [52]. A 4.5-times increase in the expression of cell-bound RAGE and 2.3-times increase in the gene expression of AGE Receptor 1 were noted in periodontitis-affected sites. A significant increase in AGE Receptor 1 gene expression was noted in periodontitis-affected sites compared to unaffected sites [52]. The levels of sRAGE was less in T2DM subjects with periodontitis (GCF: 442.425 ± 72.88 pg/mL; serum: 460.23 ± 81.23 pg/mL) compared to those without periodontitis (GCF: 536.88 ± 78.83 pg/mL, serum: 555.99 ± 83.53 pg/mL). The sRAGE levels in GCF (607.56 ± 84.40 pg/mL) and serum (626.565 ± 84.54 pg/mL) were found to be less in healthy subjects with periodontitis compared to those without periodontitis (GCF: 690.74 ± 68.38 pg/mL; serum 732.88 ± 68.97 pg/mL) [74].

### 3.3. Detection of AGE, Receptors of RAGE and sRAGE in Serum

The presence of periodontitis increased the serum AGE levels in both normoglycemic and hyperglycemic individuals. Healthy subjects with periodontitis had higher serum AGEs compared to healthy subjects without periodontitis (15.91 ng/mL vs. 6.60 ng/mL, respectively; mean difference = 9.317 ± 4.47; *p* = 0.041) [53]. However, the serum AGE levels in subjects with T2DM and periodontitis (29.92 ng/ml) were higher than healthy subjects with periodontitis and those without periodontitis [53]. Although this study showed that periodontitis is associated with higher AGE levels in serum, a correlation between clinical periodontal parameters (PD, CAL, BOP, GI, and plaque index) and serum AGE concentrations could not be established [53].

In contrast to the previous results, Takeda et al. reported no significant difference in the serum AGE levels of patients with T2DM and periodontitis (2.6 ± 1.0 mU/mL) compared to subjects with T2DM and no periodontitis (2.5 ± 0.8 mU/mL) [66]. However, this study reported a positive correlation between serum AGE levels and the severity of periodontal destruction as measured by the percentage ratio (%) of teeth with CAL >5 mm. Subjects with periodontal destruction in less than 10% of the teeth had lower AGE levels (2.2 mU/mL) compared to those in more than 70% of the teeth with periodontal destruction (3.3 mU/mL; *p* < 0.05) [66]. A correlation between sRAGE, esRAGE, and cRAGE levels in serum was also observed in the periodontitis [52,67]. Detzen et al. demonstrated that the serum sRAGE levels indirectly reflect the serum AGEs levels. The sRAGE levels were significantly lower in patients with periodontitis compared to subjects without periodontitis (0.95 ± 0.4 ng/mL versus 1.17 ± 0.4 ng/mL, *p* = 0.008) [52]. However, the serum levels of esRAGE were similar in the T2DM patients with or without periodontitis (periodontitis: 0.29 ± 0.15 ng/mL, control: 0.30 ± 0.12 ng/mL, *p* = 0.775). In contrast, Wu et al. found no statistically significant difference in the sRAGE levels in plasma in DM subjects with or without periodontitis. However, the levels of sRAGE were found to be more in healthy individuals having ‘RAGE G82G genotype’, irrespective of the presence or absence of periodontitis [67].

## 4. Discussion

The results of this review showed that inflamed periodontal tissues and periodontal pathogens add to the existing AGE levels in both normoglycemic and hyperglycemic subjects [53,54,56,69,72]. The presence of periodontitis in normoglycemic individuals can increase the AGEs levels in serum (by 2.4 times) [53], saliva (by 1.9 times) [54], and GCF (by 2.68 times) [72] compared to those without periodontitis. Moreover, T2DM patients with periodontitis will have 1.4-times more AGE levels in saliva than T2DM subjects without periodontitis [54]. The increase in AGE levels in healthy subjects with only periodontitis indicates that periodontitis can affect the AGE levels, in the absence of hyperglycemia. The presence of periodontitis adds to the exiting AGEs and increased AGE levels in turn impair glycemic control and increase insulin resistance.

Several mechanisms explain how inflamed periodontal tissues can elevate AGE levels in both normoglycemic and hyperglycemic individuals. Inflamed periodontal tissues have been recognized as ‘foci of chronic infection’ and ‘inflammatory milieu’ [76]. The interaction of the periodontal pathogens with the host receptors triggers an immune response with transendothelial migration and activation of various immune cells such as neutrophils, macrophages, lymphocytes, and fibroblasts in the gingival and periodontal tissues. The activated neutrophils and macrophages infiltrating the inflamed periodontal tissues phagocytose the pathogens and in turn release various ROS and proteolytic enzymes in the gingival connective tissue [26,42,77,78,79,80]. These ROS enter the systemic circulation and lead to the mitochondrial induced AGEs formation (Figure 1) [27,81,82,83,84,85,86,87,88,89,90]. The increase in the AGE levels increases the systemic oxidative stress, which in turn led to more glycemic load and AGE formation in a cyclic manner. The ROS species affects the insulin receptors and increase insulin resistance by affecting the molecules and enzymes of metabolism such as protein kinase C, nitric oxide synthase, and prostacyclin synthase. The impaired insulin signaling and reduced glucose uptake increase the body’s free glucose, leading to more AGE formation [91]. Increased AGEs bind to various cells and proteins in the body and impair their functions. The binding of AGE to the neutrophils impairs the neutrophil chemotaxis and phagocytosis, which will, in turn, exaggerate the inflammatory burden in the periodontal tissues. The glycation of serum albumin by increased AGEs indirectly induces the expression of TNFα, an important mediator inhibiting insulin signaling and causing more AGE production [92]. TNFα can even increase the process of glycogenolysis and impair glucose uptake, which in turn increases the blood glucose level leading to hyperglycemia and increased AGE levels [41,93]. Periodontitis-induced free radicals and proinflammatory cytokines (IL6, IL17, TNF, CRP) can even activate the hepatocytes and increase CRP production, increasing the systemic inflammatory burden and AGE formation. IL6 and IL17 have also been shown to induce apoptosis of beta cells of the pancreas, reduce insulin secretion, and alter the GLUT2 receptor. These mechanisms impair the glucose uptake and increase the blood glucose levels, subsequently increasing AGE formation [15,94,95,96,97,98,99]. These inflammatory mediators also affect lipoprotein lipase activity and increase the serum lipid levels (hyperlipemia). Hyperlipemia increases AGE precursors’ lipoxidation and glycation and leads to more AGE formation [100]. Periodontitis stimulates the release of adipokines from adipose tissues, which impairs the metabolic control resulting in hyperglycemia, and hyperlipidemia, which in turn affects the AGE levels [82,101,102]. Kashket et al. observed that *Tannerella forsythia* and *Porphyromonas gingivalis*, common periodontal pathogens, can endogenously produce AGE products in the inflamed gingival and periodontal tissues [62,66,71,103]. *Tannerella forsythia* was found to produce methylglyoxal synthase (MgsA), an enzyme that catalyzes the formation of methylglyoxal, a dicarbonyl intermediate and a precursor of AGEs [104]. Methylglyoxal was observed to covalently binds to arginine and lysine residues in the gingival connective tissue to form various AGE adducts [71]. *P. gingivalis* has even been shown to induce insulin resistance via formation branched chain amino acid synthesis [103].

Based on this evidence, periodontitis should be considered a plausible cause of altered AGE levels in both normoglycemia and hyperglycemia. Since individuals with DM have an increased rate of AGE formation and risk of various microvascular and microvascular complications because of AGE deposition, any factor that adds to the exiting burden of AGE should be carefully evaluated and corrected. Existing literature emphasizes only the role of hyperglycemia-induced AGE and how it affects the health of oral tissues. However, it is also crucial to note that periodontitis, diabetes, and AGE form a vicious cycle. This evidence could explain the role of periodontitis in the development of incident DM, prediabetes, impaired metabolic control and insulin resistance, and worsening HbA1c levels in T2DM patients [15,56,59,97,103,104,105,106,107,108]. The link between periodontitis and AGE also explains the improvement seen in the glycated hemoglobin (HbA1c), ROS and blood glucose levels by non-surgical periodontal therapy (scaling and root planing) [40,41,109,110,111,112,113,114].

However, one should note that the results of this review are based on heterogenic and cross-sectional studies. One of the main limitations of the included studies is that there are no standardized criteria or method to diagnose T2DM and periodontal disease. There are variations in the amount of PD and CAL for defining periodontitis and non-periodontitis groups. Hence, based on the heterogenicity of the study design, it is difficult to deduce the exact change in the AGE levels induced by periodontitis alone. For instance, one study showed a correlation between the periodontitis and serum AGE levels [53]. A second study could not find any changes in AGE levels in patients with T2DM and periodontitis compared to patients with T2DM alone [66]. However, this difference may be partly explained by difference in the diagnostic criteria for selecting patients with periodontitis, varying severity of periodontitis, and glycemic load at baseline and difference in the study population. Additionally, there is a variation in the duration of DM and periodontitis in the studies included in this review. Since AGE levels accumulate over time, comparisons between patients diagnosed with DM and those who have had DM for a long time cannot be made. Furthermore, it should be noted that since elevated serum AGE levels in patients with diabetes can, in turn, modulate the severity and progression of the periodontitis, the current evidence is not enough to comment on the directions of the reported observations. Hence, we cannot confirm the outset and source of increased AGE formation and the sequential pathogenic mechanism of how periodontitis modulates the AGE levels. Moreover, the local increase in the AGE levels in saliva, GCF, and gingival tissues in both normoglycemic and hyperglycemic subjects with periodontitis can be linked to the periodontal pathogens forming methylglyoxal, a precursor of AGE, or to the interaction of excess glucose in the blood with the proteins in the gingival connective tissues. However, in normoglycemic patients, the elevated levels of AGEs in saliva and GCF—which are transudates of serum—clearly points towards inflamed tissues’ capability to reproduce AGEs. This evidence warrants further studies to confirm how oral tissues produce AGEs. It also underpins the large body of evidence that inflamed periodontal tissues can act as a risk factor for diabetes. Its therapy is important to control the blood glucose levels in T2DM subjects- and its associated complications. The current evidence lacks information about the molecular composition of AGEs produced in periodontal tissues of normoglycemic subjects compared to those made in hyperglycemic patients. Therefore, there is an urgent need for well-designed studies in larger populations with standardized criteria for differentiating patients with and without periodontitis to draw definitive conclusions.

## 5. Conclusions

Inflamed periodontal tissues add to the systemic AGE levels in both normoglycemic and hyperglycemic individuals. The presence of periodontitis increases the AGE levels in serum, saliva, GCF, and gingival tissues. The combined effect of DM and periodontitis on the AGE levels is higher compared to DM and periodontitis alone. However, since this evidence is based on cross-sectional study designs, future experimental studies are warranted to explore AGE’s specific nature and concentrations produced by inflamed periodontal tissues. Additionally, prospective studies on animal models should explore the role of periodontal pathogens in initiating prediabetic conditions. If the initiation of periodontitis and invasiveness of a specific periodontal pathogen is found to trigger insulin resistance, increase systemic AGE levels, and cause the development of a pre-diabetic stage, it would provide an important and causal inference on the role of periodontitis on AGE formation, insulin resistance, and T2DM.

## Figures and Tables

**Table 1:** Search strategy for different databases for including articles for the title and abstract screening.

S/No	Database	Search String Used	Total Number of Articles
1.	**PubMed**	[“periodontitis” OR “periodontal disease” OR “Chronic periodontitis” OR “periodontal Inflammation”) AND (“diabetes” OR “diabetes mellitus” OR “Type 2 DM” OR “Type 2 diabetes mellitus” OR “non-insulin dependent diabetes mellitus” OR “Hyperglycemia” OR Insulin resistance)) AND (“Advanced glycation end products” OR “Advanced glycosylation end products” OR “Millard Reaction” OR “carboxymethylysine” OR “Pentosidine”)	**119**
2.	**Scopus**	“Periodontitis” OR “Periodontal disease” OR “Chronic periodontitis” OR “Periodontal Inflammation”) AND (“Diabetes” OR “Diabetes mellitus” OR “Type 2 DM” OR “Type 2 diabetes mellitus” OR “non-insulin-dependent diabetes mellitus” OR “Hyperglycemia” OR “Insulin resistance”) AND (“Advanced glycation end product” OR “Advanced glycosylation end products” OR “Millard Reaction” OR “carboxymethylysine” OR “Pentosidine”)	**958**
3.	**Web of Science**	TI = (“Periodontitis” OR “periodontal disease” OR “Chronic periodontitis” OR “periodontal Inflammation”) AND (“diabetes” OR “diabetes mellitus” OR “Type 2 DM” OR “Type 2 diabetes mellitus” OR “non-insulin dependent diabetes mellitus” OR “Hyperglycemia” OR Insulin resistance)) AND (“Advanced glycation end products” OR “Advanced glycosylation end products” OR “Millard Reaction” OR “carboxymethylysine” OR “Pentosidine”)	**6**
4.	**Dentistry and Open Science (EBSCO)**	TI (Periodontitis OR periodontal disease OR Chronic periodontitis OR periodontal Inflammation AND Diabetes OR diabetes mellitus OR Type 2 DM OR Type 2 diabetes mellitus OR non-insulin dependent diabetes mellitus OR Hyperglycemia OR Insulin resistance AND Advanced glycation end products OR Advanced glycosylation end products OR Millard Reaction OR carboxymethylysine OR Pentosidine)	**5**
5.	**Cochrane Database**	Periodontitis OR periodontal disease OR Chronic periodontitis OR periodontal Inflammation in Title Abstract Keyword AND Diabetes OR diabetes mellitus OR Type 2 DM OR Type 2 diabetes mellitus OR non-insulin dependent diabetes mellitus OR Hyperglycemia OR Insulin resistance in Title Abstract Keyword AND Advanced glycation end products OR Advanced glycosylation end products OR Millard Reaction OR carboxymethylysine OR Pentosidine in Title Abstract Keyword—with Publication Year from 2000 to 2020, in Trials	**6**
6.	**Clinicaltrials.gov**	Periodontitis and advanced glycation End products	**46**
		**Total search**	**1140**

**Table 2:** Characteristics of the included studies.

**Author/Year/Country of Origin/Study Design**	**Male/Female Ratio, AGE Range or Mean Age**	**Diagnostic Criteria for Periodontitis**	**Diagnostic Criteria for Diabetes**	**Method Used to Analyze AGE or Its Receptors**	**Groups in the Study and Sample Size in Each Group** **(*n*)**	**Outcome Analyzed** **Mean PD, CAL, BOP, and AGE Levels**				
						**AGEs/RAGE Levels**	**Any Forms of RAGE to Its Receptor**	**PD (in mm)**	**CAL (in mm** **)**	**BOP (%)**
**Serum**										
**Takeda et al. (2006) [66]** **Japan** **Analytical** **cross-sectional**	Male:Female: 1.2:1.0. Age range: 20–35 years (mean age: 57.8–12.1 years)	Presence or absence of BOP with CP: CAL > 5 mm. Periodontal health: CAL in a tooth in <5 mm	World Health Organization (not specified)	ELISA	DM + PD *n* = 69	2.5 ± 0.8 mU/mL	-	-	-	3.3 ± 4.8
					DM + no PD *n* = 28	2.6 ± 1.0 mU/mL	-	-	-	1.4 ± 2.8
**Hussein and Mohammed (2020) [53]** **Iraq** **Analytical cross-sectional**	Only male patients. Age range: 35–55 years	Periodontitis: CAL ≥ 5 mm Periodontal health: no CAL	HbA1c > 9%	ELISA	DM + PD *n* = 30	26.92 ng/mL	-	5.58 ± 0.48	5.57 ± 0.55	73.01 ± 14.60
					No DM + PD *n* = 30	15.91 ng/mL	-	5.24 ± 0.64	5.14 ± 0.41	70.40 ± 9.27
					No DM + No PD *n* = 20	6.60 ng/mL	-	-	-	-
**Detzen et al. (2019) [52]** **USA** **Case-control study**	Only male patients (100 males in each group). Mean age: 42.9 ± 9.9 years	Periodontitis group: ≥5 mm PD along with CAL ≥ 3 mm, and BOP ≥ 30% of their surfaces. Periodontal healthy: no sites with ≥5 mm PD and CAL attachment loss of < 3 mm	Normoglycemic patients	ELISA/Immunohistochemistry/spectrophotometry	No DM + PD *n* = 50	sRAGE levels = 0.95 ± 0.43 ng/mL esRAGE: 0.29 ± 0.15 ng/mL cRAGE: 0.66 ± 0.31 ng/mL	4.5-fold increase in the expression of cRAGE and 2.3 folds increase in AGER1 expression in periodontitis-affected sites compared to control	3.0 ± 1.0	2.3 ± 1.6	37.7 ± 26.6
					No DM + No PD *n* = 50	sRAGE = 1.17 ± 0.40 ng/mL esRAGE: 0.30 ± 0.12 ng/mL cRAGE: 0.88 ± 0.79 ng/mL		-	-	-
**Wu et al. (2015) [71]** **Taiwan** **Case control study**	Male: Female: DM + PD: 1:1 DM + no PD: 1:1 No DM + PD: 7.7:1.0 No DM + No PD: 1.8:1.0 Mean age: 42.1 ± 6.4 to 44.7 ± 6.1 years	Periodontitis group: CPI score of 0, 1 or 2: Non periodontitis group Periodontal healthy: CPI score 3 or 4: CP group	Individuals with DM and healthy controls	ELISA	DM + PD *n* = 172	sRAGE with the G82G genotype in plasma: 889.5 ± 493.3	-	-	-	-
					DM + NO PD *n* = 58	sRAGE in plasma with the G82G genotype: 991.3 ± 507.6 pg/mL	-	-	-	-
					No DM + PD *n* = 202	sRAGE in plasma with the G82G genotype: 845.1 ± 335.0 pg/mL	-	-	-	-
					No DM + No PD *n* = 62	sRAGE in plasma with the G82G genotype: 890.5 ± 326.4 pg/mL		-	-	-
**Serum and Gingival Crevicular Fluid**										
**Singhal et al., 2016 [74]** **India** **Analytical cross-sectional**	Male:Female: 1:1 Age range: 25–42 years	Periodontitis group: CAL ≥ 3 mm, GI > 1, PD ≥ 5 mm, and radiographic bone loss. Periodontal health: PD ≤ 3 mm, GI = 0, CAL = 0, and HbA1c ≤ 7%) and no radiographic evidence of bone loss	DM = HbA1c ≤ 7% (minimum 5 years of DM)	ELISA	DM + PD *n* = 20	-	sRAGE (GCF): 442.425 ± 72.88 pg/mL sRAGE (Serum): 460.23 ± 81.23 pg/ml	2 ± 0.655	5.60 ± 0.88	-
					DM + no PD *n* = 15	-	sRAGE (GCF): 536.88 ± 78.83 pg/mL sRAGE (serum): 555.99 ± 83.53 pg/ml	6.60 ± 1.27	0	-
					No DM + PD *n* = 20	-	sRAGE (GCF): (607.56 ± 84.40 pg/mL sRAGE (serum): 626.565 ± 84.54 pg/mL	6.15 ± 1.09	5.05 ± 1.67	-
					No DM + No PD *n* = 15	-	sRAGE (GCF): 690.74 ± 68.38 pg/mL sRAGE (serum) 732.88 ± 68.97 pg/mL	2 ± 0.66	0	-
**Akram et al. (2020) [72]** **Kingdom of Saudi Arabia** **Analytical cross-sectional**	DM + PD = Male:Female: 5.4:1.0 Mean age: 55.2 yearsNo DM + PD = Male:Female: 4.1:1.0 Mean Age: 51.5 years No DM + No PD: Male/Female:6.75:1.0 Mean age: 50.7 years	Periodontitis group: Presence of BOP, Pl, PD ≥ 4 mm, CAL ≥ 3 mm, and MBL ≥ 3 mm at six sites per tooth at least 30% of sites. Periodontal health: not mentioned	Normoglycemic→ HbA1c levels of ≤5.6% Hyperglycemic → HbA1c levels of ≥ 5.6%	GCF	DM + PD *n* = 32	521.9 pg/mL	-	49.2% (*p* < 0.001)	-	5.2% (3.5 to 5.8%) (*p* < 0.001)
					No DM + PD *n* = 31	234.84 pg/mL	-	34.4% (*p* < 0.01)	-	3.3% (3 to 5%)
					No DM + No PD *n* = 31	87.2 pg/mL	-	0% (*p* < 0.001)	-	0.8% (0 to 1.5%)
**Kashket et al., (2003) [71]** **USA** **Analytical cross sectional**	Male: Female ratio: No PD: 2.5:1.0 Mean Age: Healthy: 45.67 ± 5.7 years Male:Female ratio in PD group: 0.4:1.0 Mean age: 45.37 ± 13.1 years	Periodontitis group: minimum of four teeth with pocket depths or attachment loss of 4 mm	Normoglycemic patient (not mentioned about the glycemic control)	GCF	PD with disease sites *n* = 14	MG levels: 200.7 ± 243.1 pmol/sites	-	Mean PD with sites from which MG was collected: 5.77 ± 0.7 mm	3.27 ± 0.8 mm	24 ± 13
					PD with Healthy sites *n* = 14	MG levels: 140.9 ± 236.6 picomoles/sites	-	Whole mouth PD with sites from which MG was collected: 2.77 ± 0.6 mm	3.27 ± 0.8 mm	
					Healthy controls *n* = 7	Mean MG levels: 11.5 ± 4.4	-	Mean PD with sites from which was collected: 2.77 ± 0.5 mm	2.07 ± 0.4	10 ± 10
**Gingival Tissues**										
**Katz et al., 2005 [57]** **Analytical cross-sectional**	Male:female: Not mentioned Mean age or age range: Not mentioned	Generalized periodontal disease: CAL 30% with BOP Patients with active periodontal inflammation Periodontal health: not mentioned	Fasting blood glucose levels of <126 mg/dl as reported by the patient for without type 2 diabetes mellitus and patients with diabetes mellitus > 126 mg/dL	Immunohistochemistry, m-RNA expression and PCR	DM + PD *n* = 8	The RAGE increased in the spinous and basal layer of the inflamed gingival epithelium in subjects with and without T2DM having periodontitis. Approximately 50% increase in RAGE mRNA was noted in the gingiva of patients with DM with periodontitis compared having healthy controls with periodontitis (*p* < 0.05).				
					No DM + PD *n* = 14					
**Rajeev et al., 2011** **India [73]** **Analytical cross-sectional**	Male: Female: Not mentioned Mean age: not mentioned	Periodontal disease consisting of a PD of ≥ 3 mm and CAL of ≥6 mm Periodontal health: not mentioned	World Health Organization (b) moderately controlled patients with diabetes mellitus and 6–8% HbA1c	Immunohistochemistry	DM + PD *n* = 19	All groups showed mild to strong immunoreactivity for RAGE receptor. The patients with T2DM and chronic periodontitis had more BOP, PD, and CAL compared to the control group (*p* < 0.01). A positive correlation between RAGE receptor activation, age and HbA1c were noted.				
					No DM + PD *n* = 18	Six patients showed mild immune reactivity for RAGE. Twelve showed negative immune reactivity for RAGE.				
**Abbass et al., 2012 [56]** **U.S.A)**(break/**Analytical cross-sectional**	Male: Female: 1.2: 1.0 Mean age: 53–60 years	Periodontitis: presence of at minimum 5 sites with ≥4 mm; horizontal alveolar bone loss noted in the radiographs. PD and CAL	DM: HbA1c ≥ 6.5%No DM: HbA1c (4% to 5.9%)	Immunohistochemistry	DM + PD *n* = 15	-	46.91 b ± 5.57	6.8 ± 1.146	7.8 ± 1.424	-
					**No DM + PD** ***n* = 25**		31.42 c ± 7.42	5.8 ± 1.832	6.6 ± 1.917	-
					**No DM + No PD** ***n* = 10**	-	21.54 ± 1.46	5.8 ± 1.832	6.6 ± 1.917	
**Zizzi et al., 2013 [58]** **Italy** **Analytical cross-sectional**	DM + PD group: Male:female: 4.33:1.0 Mean age: 59 ± 1.25 years No DM + PD: Male/female: 1.28: 1.00 Mean age: 56.5 ± 1.32 yearsNo DM + No PD = Male/female:3:1 Mean Age = 55 ± 1.76 years	Generalized, severe, chronic periodontitis: more than 30% of the sites with > 5 mm CAL. Periodontal Health: PD < 3 mm, gingival index = 0 (absence of clinical inflammation) and CAL < 2 mm	Diabetes patients → No diabetes mellitus: glycated hemoglobin (HbA1c) < 6.1% and plasma glycemia lower than 100 mg/dL	Immunohistochemistry	**DM + PD** ***n* = 16**	-	Epithelium (AGE%) = 75 (65–80) Vessels (AGE%) = 63.4 ± 1.97	7 (7–7.2)	6.4 (6.3–6.6)	2 (1.4–3)
					**No DM + PD** ***n* = 16**	-	Epithelium (AGE%) =70 Vessels (AGE%) =58.7 ± 4.19	7.1	6.6	1.9
					**No DM + No PD** ***n* = 16**	-	Epithelium (AGE%) = 62.5 Vessels (AGE%) = 51.8 ± 2.88	2.6	1.1	-
**(Grimm et al., 2015) [69]** **Russia and Germany** **Cross-sectional study**	Male/female ratio: 1.22: 1.00 Age range: 40- 68 years	Periodontitis: >30% of the sites: PD > 4 mm	Not mentioned	Immunohistochemistry	**DM + PD** ***n* = 10**	Immunohistochemistry showed a 1.3-fold increase in the percentage of AGE deposition in subjects with DM (17%) compared to those without DM (13%). AGE deposition more in the vascular structures, epithelial and connective tissues’ cells.				
					**NO DM + PD** ***n* = 10**					
**Saliva**										
**Yilmaz et al., 2020 [54]** **Turkey** **Analytical cross-sectional**	% of females in each group: No DM + No PD = 17.3; No DM + PD = 15.3; DM + No PD = 33.7; DM + PD = 33.7	Periodontitis group: BOP ≥10% and interdental CAL at ≥2 non-adjacent teeth with PPD ≥ 4 mm. Periodontal health: BOP ˂ 10% of the surfaces and no sites with PPD > 3 mm besides no CAL or bone loss	Fasting plasma glucose (FPG) ≥126 mg/dL (7.0 mmol/L, and HbA1c ≥ 6.5% (48 mmol/mol)	ELISA	**DM + PD** ***n* = 63**	AGE: 332 ± 350 ng/mL	-	5.52 ± 0.53	6.29 ± 0.69	-
					**DM + no PD** ***n* = 58**	AGE: 235 ± 360 ng/mL	-	2.31 ± 0.37	2.59 ± 0.65	-
					**No DM + PD** ***n* = 29**	AGE levels: 46.8 ± 52.1 ng/mL	-	5.30 ± 0.53	5.80 ± 0.72	-
					**No DM + No PD** ***n* = 28**	AGE levels: 24.4 ± 8.5 ng/mL	-	2.26 ± 0.38	2.67 ± 0.69	-

Abbreviations: DM—diabetes mellitus; AGE—advanced glycation end product; RAGE—receptor for advanced glycation end products; sRAGE—soluble receptor for advanced glycation end products; esRAGE—encapsulated receptor for advanced glycation end product; cRAGE—cleaved receptor for advanced glycation end product; GI—gingival index; HbA1c—glycated hemoglobin; BOP—bleeding on probing; PD—pocket depth; CAL—clinical attachment level; ELISA—enzyme-linked immunosorbent assay.

**Table 3:** Risk of bias (ROB) assessment using Newcastle–Ottawa Scale (NOS).

S/No	Author, Year	Selection				Comparability	Outcomes		ROB Scores
		Representativeness of the Sample:	Sample Size	Non-Respondents	Ascertainment of the Exposure (Risk Factor):	Comparability of Subjects in Different Outcome Groups Based on Design or Analysis. Confounding Factors Controlled	Assessment of Outcome:	Statistical Test	
1.	Rajeev et al., 2011 [73]	b	b	b	a	b	B	c	4
2.	Akram et al., 2020 [72]	a	b	a	a	a/b	a	a	9
3.	Abbass et al., 2012 [56]	a	b	a	a	a	B	a	7
4.	Takeda et al., 2006 [66]	a	b	a	a	a	b	a	7
5.	Katz et al., 2005 [57]	a	b	b	b	a/b	b	a	6
6.	Singhal et al., 2016 [74]	a	b	a	a	a/b	b	a	6
7.	Yilmaz et al., 2020 [54]	a	a	b	b	a/b	a	a	7
8.	Zizzi et al., 2013 [58]	a	b	a	b	a/b	b	a	7
9.	Hussein and Mohammed (2020) [53]	a	b	a	a	b	b	a	7
10.	Detzen et al., 2019 [52]	a	a	a	a	a/b	A	a	9
11.	Kashket et al., 2003 [71]	a	a	a	a	b	a	a	9
12.	Grimm et al., 2015 [69]	b	c	b	b	b	b	b	4
13.	Wu et al., 2015 [67]	a	b	b	b	a	a	a	8

## Data Availability

The data that support the findings of this study are available from the corresponding author upon reasonable request.

## Supplementary Materials

The following supporting information can be downloaded at: https://www.mdpi.com/article/10.3390/biom12050642/s1, Table S1: List of excluded studies.
