# Supplementary material for: Are Inflamed Periodontal Tissues Endogenous Source of Advanced Glycation End-Products (AGEs) in Individuals with and without Diabetes Mellitus? A Systematic Review
Source: Biomolecules. 2022 Apr 27;12(5):642. doi: 10.3390/biom12050642 (PMC9138899; doi:10.3390/biom12050642)
Supplement: Supplementary file 1 [file biomolecules-12-00642-s001.zip › biomolecules-1653571-supplementary.pdf]

Table S1. List of excluded articles upon full-text screening.

Number	Reference	Reason for exclusion
1	Maiden MF, Pham C, Kashket S. Glucose toxicity effect and accumulation of methylglyoxal by the periodontal anaerobe Bacteroides forsythus. Anaerobe. 2004 Feb;10(1):27-32.	In vitro study<br>Not related to focus question
2	Yu S, Li H, Ma Y, Fu Y. Matrix metalloproteinase-1 of gingival fibroblasts influenced by advanced glycation end products (AGEs) and their association with receptor for AGEs and nuclear factor-κB in gingival connective tissue. J Periodontol. 2012 Jan;83(1):119-26.	Not related to focus question
3	Celecová, V., Kamodyová, N., Tóthová, L., Kúdela, M. & Celec, P. Salivary markers of oxidative stress are related to age and oral health in adult non-smokers. *J. Oral Pathol. Med.* **42**, 263–266 (2013).	Not related to Outcomes
4	Settem RP, Honma K, Shankar M, Li M, LaMonte M, Xu D, Genco RJ, Browne RW, Sharma A. Tannerella forsythia-produced methylglyoxal causes accumulation of advanced glycation end products to trigger cytokine secretion in human monocytes. Mol Oral Microbiol. 2018 Aug;33(4):292-299.	In vitro study
5	Ito Y, Bhawal UK, Sasahira T, Toyama T, Sato T, Matsuda D, Nishikiori H, Kobayashi M, Sugiyama M, Hamada N, Arakawa H, Kuniyasu H. Involvement of HMGB1 and RAGE in IL-1β-induced gingival inflammation. Arch Oral Biol. 2012 Jan;57(1):73-80.	In vitro study
6	Al-Sowygh ZH, Ghani SMA, Sergis K, Vohra F, Akram Z. Peri-implant conditions and levels of advanced glycation end products among patients with different glycemic control. Clin Implant Dent Relat Res. 2018 Jun;20(3):345-351.	Analysis of AGE levels around patients with periimplantitis<br>Not related to the focus question
7	Jiang, Hui et al. Changes in advanced glycation end products, beta-defensin-3, and interleukin-17 during diabetic periodontitis development in rhesus monkeys." Experimental biology and medicine (Maywood, N.J.). 2018: 243 (8): 684-694.	Not related to PICO
8	Chang PC et al. Progression of Periodontal Destruction and the Roles of Advanced Glycation End Products in Experimental Diabetes. Journal of Periodontology, 2013; 84(3), 379–388.	Not related to PICO
